# Utilizing High-fidelity Simulators in Improving Trainee Confidence and Competency in Code Management

**DOI:** 10.1097/pq9.0000000000000496

**Published:** 2021-12-15

**Authors:** Lauren M. Tufts, Christina A. Hensley, Marie D. Frazier, Deena Hossino, Renee S. Domanico, Jeffrey K. Harris, Susan L. Flesher

## Abstract

Simulation training is central in preparing practitioners for code management that is free from patient harm.^1^ The purpose of this study was to determine if using a high-fidelity simulator in pediatric code education would improve trainee confidence and competency compared with the use of a traditional mannequin in the same setting.

**Methods:**

Fifty-third-year medical students participated in Pediatric Advanced Life Support code training, including a mock code scenario. Students were randomized into two groups and assigned to a simulator group: high-fidelity simulator (Group 1) or traditional mannequin (Group 2). To assess competency, trainees were evaluated using a checklist of required verbalized items or performed during the mock code scenario. To assess confidence, trainees completed pre- and postintervention confidence surveys, which were collected and compared.

**Results:**

Both Group 1 and Group 2 reported increased overall confidence in code management upon completion of their training. Although confidence increased universally, Group 1 reported increased confidence over that of Group 2 in three specific areas: ability to treat respiratory arrest, ability to run a code, and knowledge of the Pediatric Advanced Life Support algorithm. Group 1 also demonstrated increased competency in code management compared with Group 2 in four key code components: checking airway, checking breathing, checking pulses, and checking capillary refill.

**Conclusions:**

Trainee confidence increases after completion of Pediatric Advanced Life Support code training, regardless of simulator type utilized. However, trainees were more competent in code management when trained using a high-fidelity simulator compared with a traditional mannequin.

## INTRODUCTION

Medical education has traditionally utilized a “see one, do one, teach one” teaching style.^2–4^ Unfortunately, this teaching style lacks standardization and relies on chance patient exposure within the confines of a work-hour-restricted environment. Therefore, when this traditional teaching style is utilized alone, trainees often graduate lacking confidence and competency in common medical procedures.^5^

To offset this problematic educational structure, Resusci-Annie, the first mannequin used to teach airway management and resuscitation skills, was developed.^2,5^ Simulation training offers trainees the opportunity to experience a variety of standardized patient scenarios and practice multiple procedures. Trainees receive the benefits of hands-on learning but free of potential inadvertent patient outcomes.^3^ The concept of “the learning curve” has often accounted for increased morbidities and mortalities with inexperienced practitioners.^6^ Simulation training is a model that can avoid climbing the learning curve on actual live patients.^7^ Although only a few healthcare studies have shown improvements in patient outcomes from simulation,^8,9^ it is widely accepted that simulation and patient safety are closely connected.^3,9^ The aviation industry, as an example outside of healthcare, has a very high safety record, and the use of aviation simulators is a key factor in this success.^3,10^ As David M. Gaba (a leader in anesthesiology simulation) noted, “No industry in which human lives depend on the skilled performance of responsible operators has waited for the unequivocal proof of the benefit of simulation before embracing it.”^11^ A simulation report card related to patient safety showed strong evidence for using simulation to teach skills and emerging evidence that this reduces patient risk.^7^

Simulation-based medical training today is increasingly technologically advanced. Simulators produce realistic physical examination findings, including heart and lung sounds, pupillary dilation and constriction, cyanosis, and seizure activity. High-fidelity simulators also allow the performance of various procedures and interventions, including tracheal intubation, intraosseous line placement, urinary catheterization, umbilical catheterization, and umbilical, arterial, and venous line placement, among others. After these interventions are performed, simulators can reflect real time changes in clinical condition.

Several publications reveal improved trainee confidence following code education utilizing simulators. Many of these publications are limited to the study of resident physician trainees and only report outcomes in trainee confidence.^12^ Limited data exist on outcomes of both trainee confidence and competency, especially when the trainees are medical students.^13^

In this study, we provide medical students with pediatric code education and mock code training, utilizing both high-fidelity simulators and traditional mannequins. Several studies have looked at high versus low fidelity with conflicting results. Some found no difference in results,^14,15^ and one actually found high-fidelity led to equal or lesser performance with overconfidence.^16^ Crofts, Grady, and Rodgers found high fidelity simulation led to a better performance.^17–19^ Importantly, Mills concluded that the immersion and intensity of high-fidelity simulation created additional cognitive burden with significant educational merit.^20^ We hypothesize that both trainee confidence and competency in the management of pediatric code scenarios will increase when utilizing high-fidelity simulators compared with using traditional mannequins.

## METHODS

We developed a multidisciplinary team consisting of a fourth-year medical student, a pediatric resident physician, an attending pediatric hospitalist, an attending pediatric intensivist, an attending neonatologist, a Pediatric Intensive Care Unit (PICU) transport nurse, and a PICU clinical coordinator. This project was reviewed by Marshall University’s Institutional Review Board and granted exempt status (IRB # 883204).

Fifty ACLS-certified third-year medical students participated in our study. Training was scheduled during the first 4 weeks of the students’ 8-week pediatric block rotation. Because the pediatric block rotation lasts 8 weeks, and one educational session was held for each group of rotating students, training was performed once every 8 weeks. Our study occurred over 8 months, during which 4 training sessions were held.

We randomized participating students through a computer-generated randomization tool into one of the two training groups. If randomized to Group 1, students utilized a high-fidelity simulator (Gaumard Simulator Newborn Hal) during their code training. If randomized to Group 2, a traditional mannequin was used (Laerdal Medical Baby Anne). All trainees remained within their assigned randomized group throughout the study.

Trainees began by attending a 1-hour pediatric code lecture led by our pediatric intensivist. Our intensivist utilized the Pediatric Advanced Life Support (PALS) algorithm to teach fundamental pediatric code management. Both groups received the same lecture by the same intensivist in an attempt to offset bias. The intensivist was blinded to the group assignment of the participants.

Upon completion of the lecture, trainees participated in an additional 1-hour session of hands-on mock-code training. During this portion of training, Group 1 participants utilized their assigned high-fidelity simulator, and Group 2 utilized their assigned traditional mannequin. Trainees practiced chest compressions, tracheal intubation, and intraosseous line placement during a standardized code scenario. Both groups received the same orientation on where to listen for breath sounds, feel for pulses, etc. Group 1 was led by an attending pediatric hospitalist and an attending pediatric intensivist, as these team members were educated in operation of the high-fidelity simulator. An attending neonatologist led Group 2, along with assistance from a pediatric resident, PICU transport nurse, and PICU clinical coordinator.

**Fig. 1.**
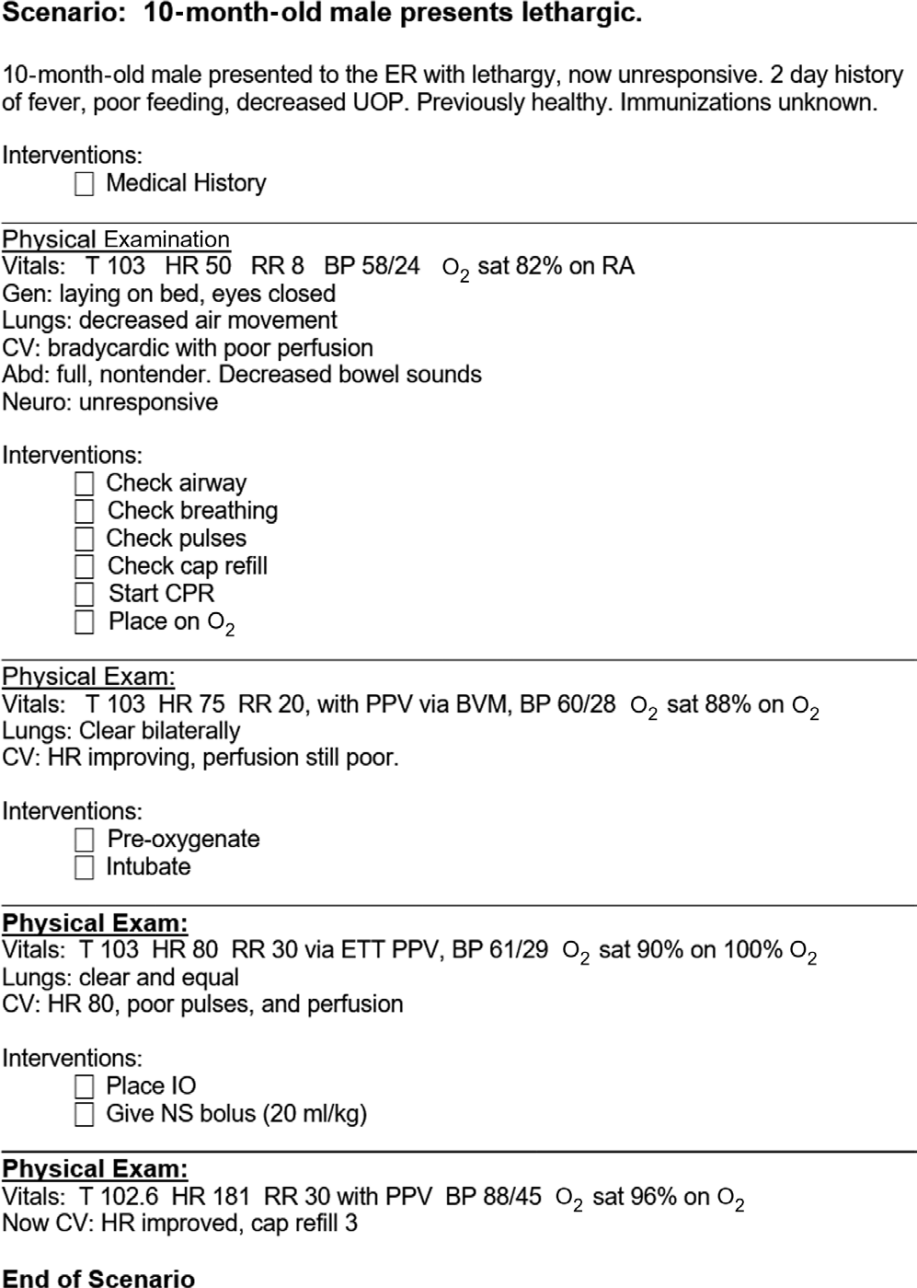
Code scenario used to measure trainee competency in code management.

Upon training completion, students were evaluated individually for competency in code management using a challenging standardized code scenario (Fig. 1). The evaluation consisted of a 11-item checklist of appropriate actions the trainees were expected to complete. Each trainee received a checkmark for an item if the appropriate action was verbalized or performed during the scenario. Trainees completed their evaluations utilizing their assigned simulator. Each group’s code competency evaluations were led by the same team members who performed their hands-on mock-code training. Both groups completed the same standardized code scenarios during their training and assessments to minimize bias.

**Fig. 2.**
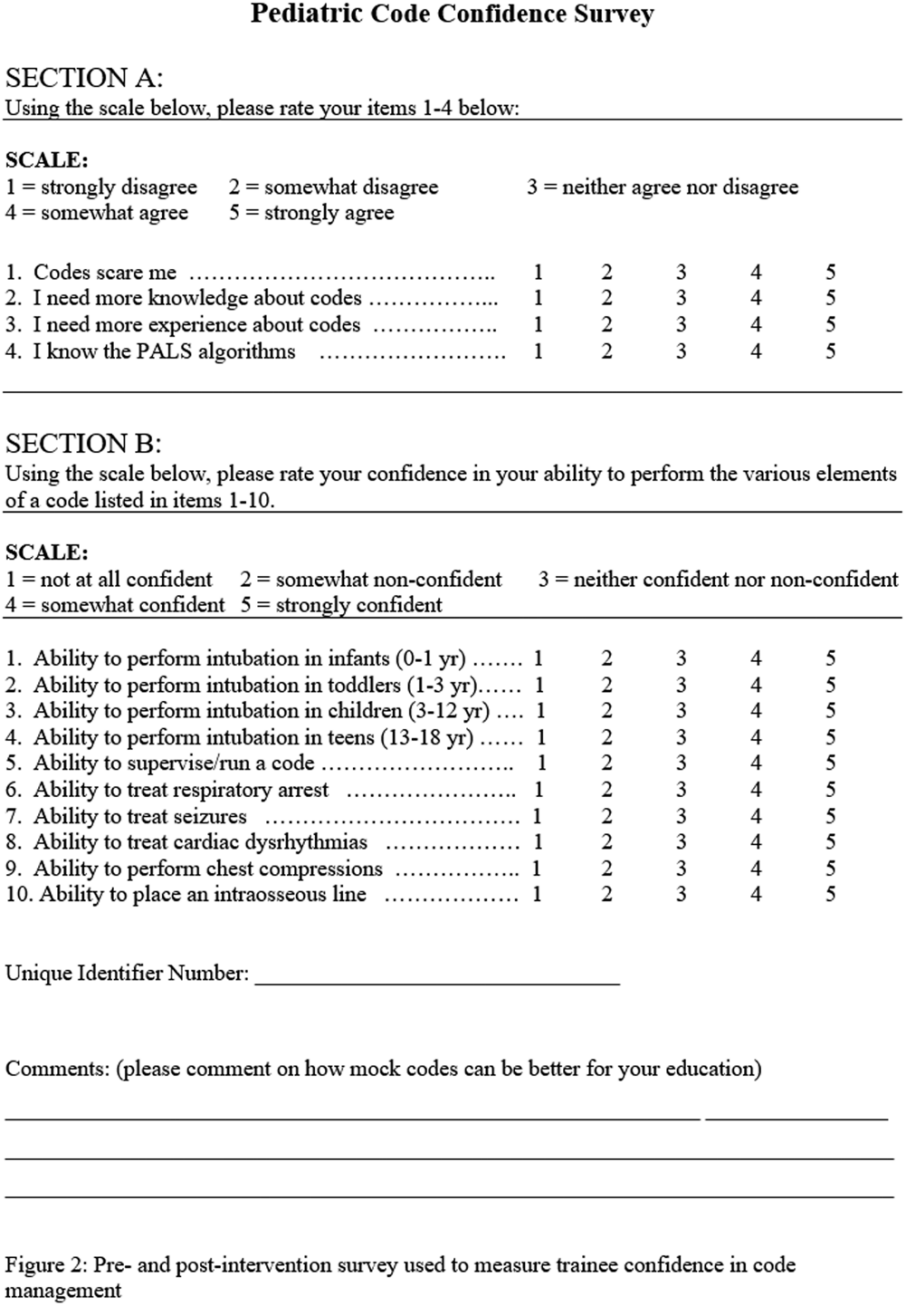
Pre- and postintervention survey used to measure trainee confidence in code management.

To measure trainee confidence in code management, students completed a pre-and postintervention confidence survey (Fig. 2). We utilized a published, nonvalidated code confidence survey by Tofil et al, based on the work of Cappella et al.^21–23^ This 14-question survey was scored on a five-point Likert scale.

Data were analyzed with GraphPad Prism (v7.03) statistical software (GraphPad Software, La Jolla, Calif.). Data from all four sessions were combined to create one high-fidelity group and one low-fidelity group for analysis. Competency evaluation results were tested between the two different simulator groups, using the Fisher’s exact test and 95% confidence interval. In doing so, we evaluated trainee competency after completion of code education, based on the simulator utilized (Table 1). Pre- and post-code education survey questions were first analyzed within each group using a Wilcoxen signed rank test with a 95% confidene interval (Table 2). This evaluated the change in confidence within each group. To evaluate the difference in confidence between the two groups, both pre- and post-code education survey questions were analyzed using the Mann-Whitney rank-sum test and a 95% confidene interval (Table 3). We chose to use nonparametric tests for these analyses for their more conservative nature. We could not assume that our data had a normal distribution due to the type of survey used.

**Table 1.** Fisher Exact Test Comparing Completion of Scenario Check-off Components in Group 1 versus Group 2

Component	Group 1 [n = 27] (% Completed)	Group 2 [n = 23] (% Completed)	*P*
Medical history	21 (77.8)	21 (91.3)	0.2609
Check airway	27 (100.0)	12 (52.2)	**<0.0001**
Check breathing	27 (100.0)	19 (82.6)	**0.0384**
Check pulses	26 (96.3)	11 (47.8)	**0.0002**
Check capillary refill	21 (77.8)	5 (21.7)	**0.0002**
Start CPR	26 (96.3)	23 (100.0)	>0.9999
Place on oxygen	26 (96.3)	23 (100.0)	>0.9999
Pre-oxygenate	26 (96.3)	22 (95.7)	>0.9999
Intubate	27 (100.0)	22 (95.7)	>0.9999
Place intraosseous	27 (100.0)	23 (100.0)	>0.9999
give saline bolus	27 (100.0)	23 (100.0)	>0.9999

Bold values are statistically significant.

**Table 2.** Intra-group Wilcoxon Signed Rank Comparison, Reporting Median (Inter Quartile Range (IQR))

Survey Questions	Group 1 [n = 27]			Group 2 [n = 23]		
	Pre-training Median (IQR)	Post-training Median (IQR)	*P*	Pre-training Median (IQR)	Post-training Median (IQR)	*P*
Codes scare me	4.00 (3.00, 5.00)	2.00 (2.00, 4.00)	**0.0007**	4.00 (3.00, 4.00)	3.00 (2.00, 4.00)	**0.0327**
Need more knowledge about codes	5.00 (4.00, 5.00)	3.00 (3.00, 4.00)	**<0.0001**	5.00 (4.00, 5.00)	4.00 (2.00, 4.00)	**<0.0001**
Needs more experience with codes	5.00 (5.00, 5.00)	4.00 (4.00, 4.00)	**0.0001**	5.00 (4.00, 5.00)	4.00 (3.00, 4.00)	**0.0006**
I know the PALS algorithms	2.00 (1.00, 2.00)	4.00 (4.00, 4.00)	**<0.0001**	1.00 (1.00, 2.00)	3.00 (2.00, 4.00)	**<0.0001**
Ability to perform intubation infants (0–1 y)	2.00 (1.00,2.00)	4.00 (4.00, 4.00)	**<0.0001**	2.00 (1.00, 3.00)	4.00 (3.00, 4.00)	**<0.0001**
Ability to perform intubation toddlers (1–3 y)	2.00 (1.00, 3.00)	4.00 (3.00,4.00)	**<0.0001**	2.00 (1.00, 3.00)	4.00 (3.00, 4.00)	**<0.0001**
Ability to perform intubation children (3–12 y)	2.00 (1.00, 3.00)	4.00 (3.00, 4.00)	**<0.0001**	2.00 (1.00, 3.00)	4.00 (3.00, 4.00)	**<0.0001**
Ability to perform intubation teens (13–18 y)	2.00 (1.00, 3.00)	4.00 (4.00, 4.00)	**<0.0001**	2.00 (1.00, 3.00)	4.00 (3.00, 4.00)	**<0.0001**
Ability to supervise/ run code	1.00 (1.00, 2.00)	3.00 (3.00, 4.00)	**<0.0001**	1.00 (1.00, 2.00)	3.00 (1.00, 4.00)	**0.0005**
Ability to treat respiratory arrest	2.00 (1.00, 2.25*)	4.00 (4.00, 4.00)	**<0.0001**	2.00 (1.00, 3.00)	3.00 (3.00, 4.00)	**<0.0001**
Ability to treat seizures	2.00 (2.00, 3.00)	3.00 (2.00, 3.00)	**0.0002**	2.00 (1.00, 3.00)	3.00 (2.00, 4.00)	**0.0013**
Ability to treat cardiac dysrhythmias	2.00 (2.00, 3.00)	3.00 (3.00, 4.00)	**0.0049**	2.00 (1.00, 3.00)	3.00 (2.00, 4.00)	**0.0003**
Ability to perform chest compressions	4.00 (3.00, 5.00)	5.00 (4.00, 5.00)	**0.0010**	4.00 (3.00, 5.00)	5.00 (4.00, 5.00)	**0.0071**
Ability to place intraosseous line	1.00 (1.00, 2.00)	4.00 (4.00, 5.00)	**<0.0001**	1.00 (1.00, 2.00)	4.00 (3.00, 5.00)	**<0.0001**

Bold values are statistically significant.

*One student did not answer this question in Group 1.

**Table 3.** Inter-group Mann-Whitney Rank Sum Comparison, Reporting Median (Inter Quartile Range (IQR))

Survey Questions	Pre-training			Post-training		
	Group 1 [n = 27] Median (IQR)	Group 2 [n = 23] Median (IQR)	*P*	Group 1 [n = 27] Median (IQR)	Group 2 [n = 23] Median (IQR)	*P*
Codes scare me	4.00 (3.00, 5.00)	4.00 (3.00, 4.00)	0.7185	2.00 (2.00, 4.00)	4.00 (3.00, 4.00)	0.1237
Need more knowledge about codes	5.00 (4.00, 5.00)	5.00 (4.00, 5.00)	0.5187	3.00 (3.00, 4.00)	5.00 (4.00, 5.00)	0.3336
Needs more experience with codes	5.00 (5.00, 5.00)	5.00 (4.00, 5.00)	0.7333	4.00 (4.00, 4.00)	5.00 (4.00, 5.00)	0.7796
I know the PALS algorithms	2.00 (1.00, 2.00)	1.00 (1.00, 2.00)	0.8990	4.00 (4.00, 4.00)	1.00 (1.00, 2.00)	**0.0334**
Ability to perform intubation infants (0–1 y)	2.00 (1.00,2.00)	2.00 (1.00, 3.00)	0.5540	4.00 (4.00, 4.00)	2.00 (1.00, 3.00)	0.2262
Ability to perform intubation toddlers (1–3 y)	2.00 (1.00, 3.00)	2.00 (1.00, 3.00)	0.8938	4.00 (3.00,4.00)	2.00 (1.00, 3.00)	0.3525
Ability to perform intubation children (3–12 y)	2.00 (1.00, 3.00)	2.00 (1.00, 3.00)	0.8058	4.00 (3.00, 4.00)	2.00 (1.00, 3.00)	0.3333
Ability to perform intubation teens (13–18 y)	2.00 (1.00, 3.00)	2.00 (1.00, 3.00)	0.7991	4.00 (4.00, 4.00)	2.00 (1.00, 3.00)	0.2115
Ability to supervise/run code	1.00 (1.00, 2.00)	1.00 (1.00, 2.00)	0.7172	3.00 (3.00, 4.00)	1.00 (1.00, 2.00)	**0.0239**
Ability to treat respiratory arrest	2.00 (1.00, 2.25*)	2.00 (1.00, 3.00)	0.6343	4.00 (4.00, 4.00)	2.00 (1.00, 3.00)	**0.0125**
Ability to treat seizures	2.00 (2.00, 3.00)	2.00 (1.00, 3.00)	0.4724	3.00 (2.00, 3.00)	2.00 (1.00, 3.00)	0.7665
Ability to treat cardiac dysrhythmias	2.00 (2.00, 3.00)	2.00 (1.00, 3.00)	0.1525	3.00 (3.00, 4.00)	2.00 (1.00, 3.00)	0.4245
Ability to perform chest compressions	4.00 (3.00, 5.00)	4.00 (3.00, 5.00)	0.8878	5.00 (4.00, 5.00)	4.00 (3.00, 5.00)	>0.9999
Ability to place intraosseous line	1.00 (1.00, 2.00)	1.00 (1.00, 2.00)	0.5502	4.00 (4.00, 5.00)	1.00 (1.00, 2.00)	0.1023

Bold values are statistically significant.

*One student did not answer this question in Group 1.

## RESULTS

Of the 50 participating medical students, 27 students were randomized to Group 1 and 23 students were randomized to Group 2. Data of trainee competency revealed improved competency in Group 1 over that in Group 2, with statistically significant differences in four components of the scenario checklist (Table 1).

Data of trainee confidence revealed confidence improvement in both groups after training completion. This was reflected in the postcode confidence survey, which resulted in statistically significant improvements in every survey question within each group when compared with the pre-code education survey for that group (Table 2). Although both groups reported increased confidence, Group 1’s confidence improved more than that of Group 2 in three of the surveyed areas (Table 3).

## DISCUSSION

Physicians are expected to enter the setting of a patient code confident in their knowledge of appropriate interventions and competent in their ability to carry out those interventions. Because patient codes are unpredictable, it is essential that physicians receive appropriate training for adequate preparedness in these situations. However, pediatric codes are more infrequent than adult codes, and residents and students train under restricted work hours. Therefore, there is less opportunity for education through participation in live codes. This makes education through mock-code scenarios essential. Not only can residents and students learn and practice life-saving code interventions and procedure techniques, but they can do so without jeopardizing patient safety in the process.

This education, however, is appropriate only if it produces more competent and confident trainees. Studies previously identified improved confidence in resident physicians after code training completion. However, data on trainee competence and the effect of training on medical students are lacking. Therefore, this study aimed to determine if completion of code training utilizing a high-fidelity simulator would improve medical students’ confidence and competence compared with the use of a traditional mannequin.

Our results indicate that completion of code training improved medical students’ overall confidence in pediatric code management, regardless of the simulator used. However, confidence in the ability to treat respiratory arrest, ability to run a code, and knowledge of PALS algorithms was in higher in the high-fidelity simulator group than in the traditional mannequins group. We believe perhaps the didactic teaching about these three items was better reinforced by using a more life-like mannequin, whereas other confidence items such as placement of an IO did not result in a different experience in the high-fidelity versus traditional groups. Although it is unlikely that a medical student would be running a code, we believe their perceived increased confidence speaks well of the value of their learning experience. Fortunately, this increased confidence was accompanied by increased competence in medical student code management concerning checking airway, checking breathing, checking pulses, and checking capillary refill using high-fidelity simulators compared with traditional mannequins. These four elements are essential assessment skills reinforced when learning in an environment that is truly representative of changes in a child during physical deterioration. Our study appears to show that learners who have practiced and seen subtle changes can improve short-term competence and confidence. Further research is needed to determine if they will translate these assessment skills to real-life management, allowing them to provide safer patient care.

High-fidelity simulators provide advantage over traditional mannequins, as simulators produce a life-like patient with a real-time change in clinical condition. This creates an educational environment that closely mimics a live pediatric code. For example, it seems that actually seeing the mannequin become apneic and cyanotic impacts the learner differently than just being told, “Your patient has stopped breathing, and the oxygen saturation has dropped.” We believe this authentic replication underlies improved trainee competence and confidence. With the literature being controversial regarding high versus low fidelity, our study provides another important piece in addressing this knowledge gap. Interestingly, a consistent finding in literature is that there is a preference for the use of high-fidelity simulation.^24^ Perhaps learner preference for being taught using high-fidelity simulation may be a factor in improving performance because of increased engagement in the learning process.

This study evaluated medical student confidence and competence immediately following completion of code training utilizing either a high-fidelity simulator or a traditional mannequin. We did not re-evaluate trainee confidence and competence and, therefore, cannot report data on the sustainment of these improvements in code management. Prior studies report that CPR skills begin to diminish as quickly as 2 weeks after training and continue to decline, reaching pretest levels by 1–2 years post-training.^22,23,25^ Therefore, we see that maintaining trainee confidence and competence in code management is necessary and an opportunity for future research.

In addition to the lack of addressing sustainability, our study has several limitations. While the same individual provided the initial 1-hour pediatric code lecture to each group, different individuals provided the second hands-on 1-hour training session opening the possibility of different teaching styles and abilities. Furthermore, the selection of instructor choice was based on familiarity with operating the high-fidelity simulator, but the assignment may not have been equitable because neonatologists use PALS less often than intensivists and hospitalists. There was no analysis to assess interrater reliability for competency evaluation between evaluators for Group 1 and Group 2. This was partially mitigated by the items for evaluation that were entirely objective; for example whether or not someone started CPR or placed oxygen was a binary yes or now.

The confidence survey, while previously published, was not validated. Even though we found statistically significant results, they may not be clinically relevant. The study was performed in a simulation setting only, and further study is needed to reveal how real-world practice is impacted. There is also the potential for measurement bias. The students in the high-fidelity group may have expected a better experience and therefore answered the confidence questions higher on the survey.

By identifying opportunities for improvement in medical education, utilizing advanced technology within this education, and determining ways in which to sustain subsequent trainee knowledge, we are in turn working toward our global aim of safer patient care. Although we acknowledge that skills learned in a medical school will need constant reinforcement to achieve our aim, we believe that it is essential to begin training early to lay the groundwork for teamwork, communication, and situational awareness. Helping students develop these skills earlier in their training provides a benefit for future training and safer patient care in the clinical setting as resident physicians.

## DISCLOSURE

The authors have no financial interest to declare in relation to the content of this article.
